# SUPPORTING SAFE AND HEALTHY SEXUAL EXPRESSION IN LONG-TERM CARE: A REVIEW OF CANADIAN RESOURCES

**DOI:** 10.1093/geroni/igae098.2137

**Published:** 2024-12-31

**Authors:** Alisa Grigorovich, Julia Brassolotto, Alessandro Manduca-Barone, Romeo Colobong, Lori Schindel Martin, Andrea Iaboni, Sid Feldman, Pia Kontos

**Affiliations:** Brock University, St. Catharines, Ontario, Canada; University of Lethbridge, Lethbridge, Alberta, Canada; University of Lethbridge, Lethbridge, Alberta, Canada; KITE Research Institute, Toronto Rehabilitation Institute – University Health Network, Toronto, Ontario, Canada; Toronto Metropolitan University, Toronto, Ontario, Canada; University of Toronto, Toronto, Ontario, Canada; Baycrest Health Sciences, Toronto, Ontario, Canada; KITE Research Institute, Toronto Rehabilitation Institute – University Health Network; Dalla Lana School of Public Health, University of Toronto, Toronto, Ontario, Canada

## Abstract

Little is known about what educational resources are available to practitioners in Canada for supporting the safe and healthy sexual expressions of persons living with dementia in long-term care (LTC). To address this gap, we conducted a critical review of publicly available online resources in Canada. Our objectives were to identify existing resources, describe key content areas and modalities, and assess strengths and weaknesses. To find resources, we conducted a systematic Google search, targeted websites searches, and contacted experts. Resources were included if they were in English, publicly accessible (e.g., not behind a paywall), focused on support of sexual expression of persons living with dementia in LTC, and informed action at the micro level of practice and/or meso level of organizational policy. We identified 9 resources, the majority of which were text-based and aimed at supporting staff or organizations in developing policies or practices regarding assessment and intervention in non-consensual sexual behavior. Guidance on consent was narrowly focused on bioethical principles, often neglecting fluctuating capacity, assent, and the role of substitute decision-makers. Although some acknowledged that healthy sexual expression in the context of dementia should be supported, the focus was primarily on support of non-erotic intimacy (e.g., hugging vs. stroking/kissing). We also found stigmatizing language (e.g., “demented”), and cursory attention to sexual and gender diversity, and ethnically and culturally diverse content. Although the available resources provide an important foundation, there is a pressing need to develop additional resources to better support sexual expression for persons living with dementia in LTC.

